# The Confluence of Sex Hormones and Aging on Immunity

**DOI:** 10.3389/fimmu.2018.01269

**Published:** 2018-06-04

**Authors:** Melanie R. Gubbels Bupp, Tanvi Potluri, Ashley L. Fink, Sabra L. Klein

**Affiliations:** ^1^Department of Biology, Randolph-Macon College, Ashland, VA, United States; ^2^W. Harry Feinstone Department of Molecular Microbiology and Immunology, Bloomberg School of Public Health, Johns Hopkins University, Baltimore, MD, United States

**Keywords:** sex, sex hormones, immunity, autoimmunity, cancer, vaccines, immunotherapy, checkpoint blockade

## Abstract

The immune systems of post-pubescent males and females differ significantly with profound consequences to health and disease. In many cases, sex-specific differences in the immune responses of young adults are also apparent in aged men and women. Moreover, as in young adults, aged women develop several late-adult onset autoimmune conditions more frequently than do men, while aged men continue to develop many cancers to a greater extent than aged women. However, sex differences in the immune systems of aged individuals have not been extensively investigated and data addressing the effectiveness of vaccinations and immunotherapies in aged men and women are scarce. In this review, we evaluate age- and sex hormone-related changes to innate and adaptive immunity, with consideration about how this impacts age- and sex-associated changes in the incidence and pathogenesis of autoimmunity and cancer as well as the efficacy of vaccination and cancer immunotherapy. We conclude that future preclinical and clinical studies should consider age and sex to better understand the ways in which these characteristics intersect with immune function and the resulting consequences for autoimmunity, cancer, and therapeutic interventions.

## Introduction

In developed countries, the population is aging, with the number of people over the age of 65 doubling in size from 2012 to 2050 (1). In developed and even developing countries, lifespan is longer for women than men (2, 3). Both sex (i.e., biological differences between males and females) and gender (i.e., social or cultural norms that define masculine and feminine) contribute to male–female differences in mortality rates among individuals 65 years and older. Why and how the sexes differ in the incidence and progression of immune-related diseases that are either specific to advanced age or that worsen with age, such as particular infections, autoimmune disease, and cancer, has not been well studied.

Aging is associated with the development of chronic inflammation and a general reduction in immune function. The effect of sex on immune function during the aging process has not been well studied. But, some studies indicate that the innate immune system of aged females may be more inflammation-prone when compared with aged males. However, aging of the adaptive immune system may occur at a faster rate in men, when compared with women. Several diseases that are associated with age are also sensitive to changes in the immune system. Therefore, herein, we will discuss the effects of age and sex on the innate and adaptive immune systems and the contribution of sex hormones to these effects. We will also examine the functional consequences of age- and sex-related changes to immunity in the contexts of vaccination, autoimmunity, cancer, and cancer immunotherapy. We conclude that sex and age should be considered in future clinical and preclinical studies to improve our understanding and treatment of age-associated diseases.

## Age-Related Changes in Immune Function

**Figure 1:**
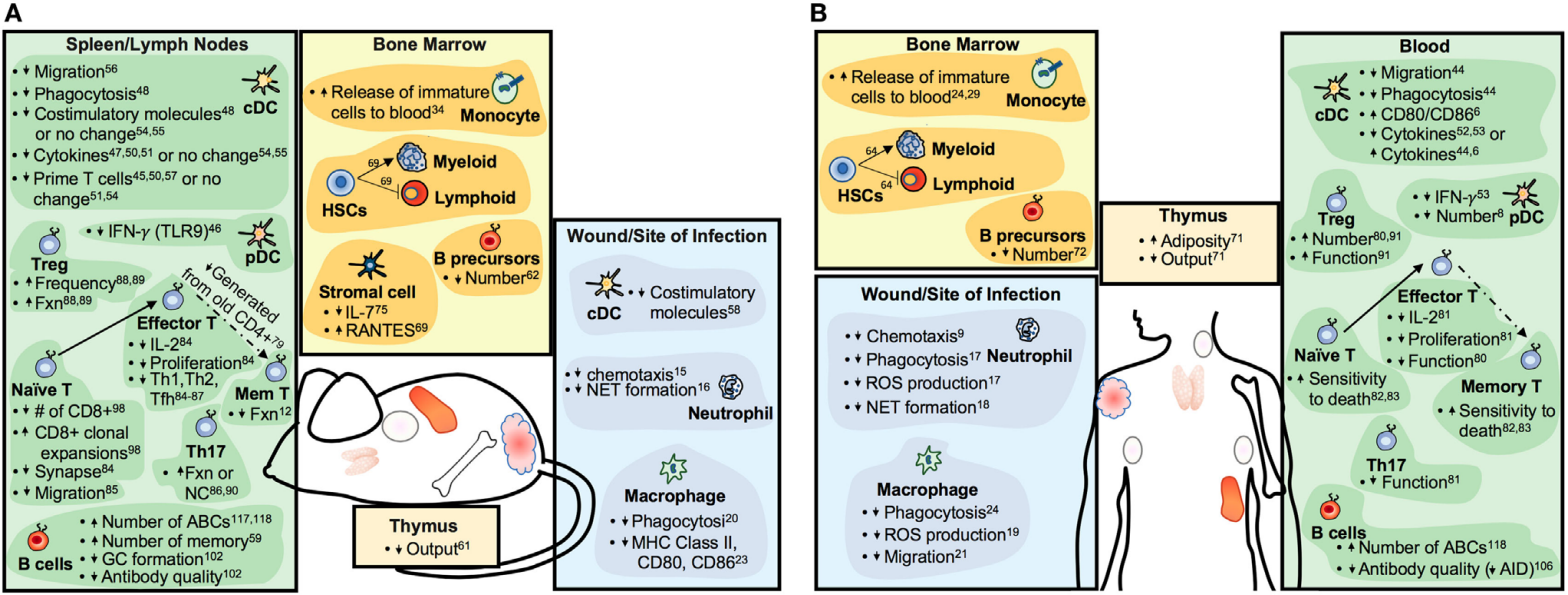
Summary of aging-related changes to the immune systems of mice **(A)** and humans **(B)**. Increases or decreases in cell numbers or particular functions are indicated by upward- or downward-pointing arrows, respectively. Abbreviations: Fxn, function; GC, germinal center; Mem, memory; ABC, age-associated B cell; NC, no change.

With age, there is a decline in the functioning of the immune system (4) that has, until recently, been assumed to occur equally in males and females. “Inflammaging,” as defined by aberrant chronic low-grade inflammatory responses, is one of the most well-characterized attributes of an aging immune system (5). The activity of dendritic cell (DC) subsets, macrophages, and neutrophils, each of which are associated with inflammation, also become altered with age (6–9). Inflammatory responses are necessary to clear pathogens and repair tissues; chronicity of inflammatory responses, however, can contribute to tissue damage and disease, especially among aged individuals. Similarly, adaptive immunity becomes less functional with age (10, 11). Reductions in lymphopoeisis along with exposure to pathogens throughout the lifespan contribute to reduced numbers of naïve lymphocytes with increased proportions of memory and memory-like lymphocytes that are associated with less robust functional outcomes (12, 13). Overall, age-associated changes to the functions of innate and adaptive immune cells (summarized in Figure 1) likely contribute to increased risk of specific autoimmune diseases and cancer, as well as altered vaccine and cancer immunotherapy efficacy.

### Age-Related Changes in Innate Immunity

Aging is associated with the secretion of pro-inflammatory cytokines, such as TNF, IL-6, and IL-1β, the cellular source of which has not yet been clearly identified (14). Innate immune cells, including DCs, neutrophils, and macrophages, become less functional and, paradoxically, more inflammatory with age. It has been difficult to determine whether systemic inflammation causes innate cell dysfunction or *vice versa*. Recent evidence discussed below suggests that inflammaging may alter the development and signaling potential of innate cells, contributing to inflammation in the absence of infection and, at the same time, a reduced ability to clear infections (15–17). Together, the elevated levels of inflammatory cytokines and diminished ability to resolve infections or local inflammation likely contribute to less functional innate responses to vaccination and increased risk of certain autoimmune diseases.

The number and proportion of plasmacytoid DCs declines during healthy aging, while frailty appears to be associated with reduced numbers of conventional DCs (8). Regardless of their number, conventional DCs from aged mice and humans migrate, phagocytose, express costimulatory molecules, secrete cytokines, and prime T cells poorly in response to exogenous antigens when compared with DCs from young conspecifics (6, 18–32). At least some of these defects appear to be cell intrinsic and related to the altered expression of toll-like receptors (TLRs) and dysregulated downstream signaling [reviewed in Ref. (33)].

Neutrophils from aged individuals have defects in accurately migrating to inflamed tissues, phagocytosing microbes, producing reactive oxygen species (ROS), and capturing microbes using neutrophil extracellular traps (9, 34–38). Like neutrophils, many functions of macrophages are negatively affected by aging including migration, phagocytosis, production of ROS and cytokines, and expression of major histocompatibility complex class II and costimulatory molecules (15, 39–43). Studies examining the cytokine response of monocytes isolated from older patients have yielded mixed results, likely due to differences in health status, isolation techniques, assay conditions, and stimuli between studies [reviewed in Ref. (33)]. Some studies have revealed a diminished ability of aged monocytes and macrophages to secrete pro-inflammatory cytokines robustly after exposure to pathogens, LPS, or other TLR ligands (44–50). Chronic exposure to inflammatory cytokines such as IL-6 and TNF-α and dysregulated expression and/or function of TLRs have been discussed as possible causes (44, 45, 48).

Several recent reports have suggested that at least in mice, inflammaging may precede and perhaps even cause dysregulation of innate immune cells, which may further contribute to inflammation. For example, aging is also associated with increased proportions of pro-inflammatory monocytes of non-classical and intermediate phenotypes (i.e., CD14^+^CD16^+^ or CD16^++^ in humans and Ly6C^hi^ in mice) that are less mature, poorer phagocytes, and may be more prone to secreting pro-inflammatory cytokines at baseline and in response to stimuli (15–17, 47, 51). In mice, aged Ly6C^hi^ monocytes both contribute to age-associated inflammation and are impaired by the inflammation with negative consequences for bacterial clearance (16). Circumstantial evidence indicates that in humans, premature migration of intermediate phenotype monocytes (CD14^++^CD16^+^) is driven by TNF-α-mediated upregulation of CCR2, as also occurs in mice (16), and may contribute to worsened disease outcomes in rheumatoid arthritis patients (52, 53).

Additional age-related changes to monocyte function may contribute to increased susceptibility to infection concomitant with a reduced ability to resolve inflammation. For example, the production of specialized pro-resolving mediators, including lipid signaling molecules produced by macrophages and monocytes, is reduced in aged mice and is associated with delayed resolution of acute inflammation (54). In addition, aged macrophages isolated from mice and humans phagocytose infectious agents and apoptotic cells less efficiently than young macrophages (15, 40, 55–59). The phagocytosis of infectious agents and apoptotic cells by macrophages is important for resolution of inflammation and restoration of tissue integrity, which is reduced with aging.

### Age-Related Changes in Adaptive Immunity

Aging is accompanied by a decline in the production of new lymphocytes as well as increased expansion and survival of antigen-specific memory lymphocytes in mice and humans (60–72). Despite reduced lymphopoeisis (73–76), the overall number of peripheral lymphocytes is maintained in aged mice (11) and humans [reviewed in Ref. (77)], with the exception of peripheral B cell numbers being reduced in older humans (78, 79). The diminished functionality of older adaptive cells may be related to age-associated changes in lymphocyte development.

The ability of aged T cells to proliferate robustly, differentiate appropriately, and generate memory is generally diminished (10, 12, 13, 80–85). However, all T cell functions are not impaired by aging. T regulatory (T_reg_) and, in some cases, T helper 17, cells increase in number and/or function with age (81, 85–93). It was recently proposed that naïve T cells produced in neonates form a long-lived, self-renewing population of “incumbent” naïve T cells that are resistant to replacement by T cells produced after the neonatal period (94). It is conceivable that accumulated damage in these long-lived incumbents may contribute to reduced naïve T cell function with age. In addition, accelerated homeostatic proliferation, as may be more likely to occur in aged individuals (95, 96), is associated with the selection of autoreactive T cells, at least in mice (97–99) and may also affect overall T cell functionality.

Changes in aged naïve T cell function likely contribute to defective memory generation and also partially explain the observation that antibodies elicited from older mice and humans are less protective compared with those from the young individuals (100–106), even though serum IgG levels increase with age in both mice and humans (107, 108). In addition, aged B cells demonstrate intrinsic defects in germinal center formation, class switch recombination, and somatic hypermutation (109–112). Aged B cells from mice and humans do not sufficiently upregulate expression of activation-induced cytidine deaminase (AID, the enzyme required for class switch recombination and somatic hypermutation) due to diminished levels of the necessary transcription factor (107, 110, 113, 114). With age, there are also more long-lived antigen-experienced B cells, including age-associated B cells (ABCs) (60, 115–120). ABCs are responsive to TLR7 and 9 ligands but less so to T cell-dependent signals and have been hypothesized to be generated by nucleic acid-containing antigens during inflammation (118, 121).

## Sex Differences in Age-Related Changes in Immune Function

Both innate and adaptive immune responses differ between males and females at young and advanced ages (summarized in Table 1). Most published studies of immune system differences between the sexes utilize young adults and do not address whether sex differences in immune function change with aging. Overall, the available data indicate that young adult females demonstrate a more reactive, inflammatory profile when compared with young adult males. A clear consensus has not emerged regarding whether these sex differences are maintained during advanced age, but the immune systems of aged women on hormone replacement therapy (HRT) and monocytes isolated from aged women, regardless of HRT status, appear to remain skewed toward an inflammatory phenotype (16, 122–124). The currently available data also indicate that the adaptive immune response of aged women may be preserved to a greater extent than in aged men. These studies are discussed in more detail below.

**Table 1:** Sex differences in innate and adaptive immune responses in young and aged individuals.

	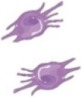 Dendritic cells	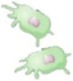 Monocytes and macrophages	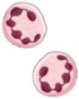 Granulocytes	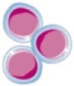 Innate lymphoid cells	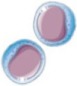 Natural killer cells	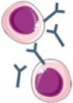 B cells	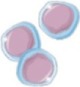 T cells
Young adults	♀ > ♂	♀ > ♂	♀ > ♂	♀ > ♂		♀ > ♂	♀ > ♂
	TLR7 activity (H)	Activation (M)	Phagocytic capacity (M)	Type 2 cytokine levels upon stimulation (M)		B cell numbers (H, M)	CD4^+^ T cell count (H, M)
	Type 1 IFN activity (H)	Phagocytic capacity (M)	Neutrophil count (M)			Antibody production (H, M)	CD4^+^/CD8^+^ T cell ratio (H)
		IL-10 production (M)	Nitric Oxide production post stimulation (H, R, M)			% switched memory B cells (H)	Activated T cell count (M)
		M2 polarization (M)					T cell proliferative capacity (M)
							Cytotoxic T cell activity (H)

	♂ > ♀	♂ > ♀	♂ > ♀	♂ > ♀	♂ > ♀		♂ > ♀
	IL-10 production (R, H)	TLR4 expression (M)			NK cell activity (R)		
		Pro-inflammatory cytokine production (M)	Neutrophil attractant chemokines (R)	Type 2 ILC count (H)	♀ = ♂		CD8^+^ T cell count (M)
		M1 polarization (M)	TLR9 expression (M)	IL-13 production upon stimulation (M)	NK cell count (H)		T_reg_ count (M)

Aged adults	♀ > ♂	♀ > ♂			♀ > ♂	♀ > ♂	♀ > ♂
	Nitric oxide synthesis (H)	CD62L, CD115 (H) expression			NK cytotoxicity (H)	Antibody production (H)	CD3^+^ T cell count (H)
	Mammalian family of mitogen-activated protein kinases (MAPK) signaling (H, M)				Immunosurveillance (H)	Age-associated B cell count (H, M)	CD4^+^ T cell count (P)
							CD4^+^/CD8^+^ T cell ratio (P) T_H_1 response (M)

	IL-15 production (H)						T_H_1 response (M)
			ND	ND			Naïve CD8^+^ T effector memory cells (p)
							T cell proliferative capacity (H, P)
		♂ > ♀					♂ > ♀
		CD38 expression (H)					CD8^+^ T cell count (P)
		Non-classical monocyte count (H)					

*Data are from studies of mice (M), rats (R), non-human primates (P), and humans (H) (125–131)*.

*ND, not determined*.

### Sex Differences in Age-Related Changes to Innate Immunity

As mentioned above, at least among young adults, innate immune responses differ between the sexes. Using murine model systems, it has been shown that the activity of pattern-recognition receptors, production of inflammatory proteins (e.g., IFN-α, IFN-γ, and TNF-α), activity of macrophages, including antigen presentation and phagocytosis is higher in females than males (132–138). Studies evaluating innate immune system differences between the sexes are scarce. But, at least one small study demonstrated that aged females display elevated concentrations of inflammatory proteins compared with males, as also occurs in young men and women (139). Several cytokines show differential levels in circulation between the sexes. For example, IL-15 is an important homeostatic cytokine in T cells, NK cell, and memory responses and is significantly upregulated in aged females when compared with age-matched males (122, 123). However, upon exclusion of individuals on HRT, such differences between sexes were no longer significant (122). After menopause, there is a significant increase in IL-1, IL-6, and TNFα, and reduction in IFNγ in women (140, 141). Testosterone has an immunosuppressive effect on inflammatory cytokine production and its decline with aging is associated with an increase in serum soluble IL-6 receptor (142). Monocyte and leukocyte subpopulations in aged males and females express different levels of receptors; males show higher CD38 expression, whereas females show higher CD62L and CD115 expression, indicating differences in their activation profiles and memory phenotypes (124). Sex differences among monocyte subsets have also been reported in aged individuals. Aged females have a higher proportion of intermediate (CD14^hi^CD16^low^) monocytes than similarly aged males, which have been shown to exhibit pro-inflammatory tendencies, as mentioned above (16, 124). Finally, NK cells in older women are superior at cancer immunosurveillance when compared with cells in older men. CD56^dim^ NK cells are more cytotoxic and more responsive to leukemic cells in aged females compared with aged males, which may explain the higher incidences of cancer in aged men compared with women in populations (143).

### Sex Differences in Age-Related Changes to Adaptive Immunity

Both humoral and cell-mediated immune responses to antigenic stimulation, vaccination, and infection are typically higher among females than males (135). Females also typically demonstrate higher basal levels of immunoglobulin (144) and higher antibody responses to viruses and vaccine antigens than males at any age (145–147). Among humans, absolute CD3^+^ T cell counts, frequencies of CD4^+^ T cells, helper T cell type 1 responses, and the ratio of CD4^+^:CD8^+^ T cells are all lower in men when compared with women (148–151).

As already mentioned, sex or gender has not traditionally been considered when evaluating age-related changes to the adaptive immune system [reviewed in Ref. (14)]. However, several groups have reported that in some ways, aging occurs at an accelerated rate in males when compared with females. For example, aged males experience a more dramatic decrease in total numbers of T and B cells and a larger increase in senescent CD8^+^ T effector memory cells that re-express the naïve marker CD45 RA (T_EMRA_) when compared with females (14, 150, 152–154). In addition, a greater proportion of aged males than females demonstrate an inverted CD4:CD8 T cell ratio, an age-related phenotype that is also associated with decreased levels of CD19^+^ B cells and CD8^+^CD28^−^ senescent T cells (152). Also, the capacity of T cells to proliferate is preserved to a greater extent in women than men throughout the aging process (154), which may be an important consideration for infectious diseases and related interventions. On the other hand, transcriptional analyses of peripheral blood mononuclear cells from aged males and females revealed several pro-inflammatory pathways, including NF-κB signaling, NO synthesis, and p38 MAPK signaling, that are reduced to a greater extent in aged females than aged males (123). Moreover, aged females have greater numbers of ABCs than young females and males of all ages (118, 119).

## The Impact of Sex Hormones on Age-Related Changes in Immune Responses

Immunological differences between males and females can arise from diverse mechanistic causes, including genetic, hormonal, and even microbiome differences between the sexes. Partly because of the ease of measuring and manipulating, sex steroids, particularly testosterone, estradiol, and progesterone, have been most well characterized as mediators of sex differences in immune responses and are the focus of this review. Sex steroids affect immune function by binding to specific hormone receptors expressed in diverse immune cells (155). With age, the hormonal milieu of females and even males changes, with an overall decline in concentrations of estrogens and progesterone in females and testosterone in males (156–158). We hypothesize that the changes in sex steroid concentrations and sex steroid receptor signaling with age may contribute to age-associated dysregulation of immune function (159). Although this has been considered in females through the comparison of pre- and post-menopausal women, few studies have considered hormonal changes in men as playing a role in age-associated changes in immune responses. Among women, with menopause, numbers of B and T cells are reduced and concentrations of IL-1β, IL-6, and TNF-α are significantly increased (141, 160, 161). Treatment of post-menopausal females with hormone replacement therapies that contain formulations of estrogen affects immune function by increasing circulating numbers of B cells and reducing baseline concentrations of pro-inflammatory cytokines when compared with post-menopausal females not on HRT (140, 161). Whether testosterone replacement therapy affects immune responses in aged human males has not been reported. In non-human primates, aged male rhesus macaques have lower frequencies of naïve CD4^+^ and CD8^+^ T cells than young males, with supplementation of androgens in aged male resulting in increased numbers of naïve T cells presumably by increasing thymic output (162). Whether treatment of aged individuals with hormone replacement therapies affects the outcome of vaccines or immunotherapies in either females or males has not been reported.

Studies in mice and humans have shown that the diversity and richness of intestinal microbiota differs between males and females after puberty, presumably due testosterone, but not estrogen (163–168). Moreover, in mice, exposure to specific microbiota at early ages also results in elevated levels of testosterone (164). Thus, testosterone appears to influence the composition of the gut microbiome and, in a positive feedback loop, specific microbes elevate testosterone levels (164). Sex-specific enrichment for particular microbes is likely to have significant influence on sex-specific immune function since particular commensals and their metabolites can dramatically modify host innate and adaptive immune function [reviewed in Ref. (169)] with serious consequences for autoimmunity, vaccine efficacy, cancer, and cancer immunotherapy [reviewed in Ref. (170, 171)]. The composition and richness of commensal microbiota is sensitive to many environmental factors as well, including diet. Importantly, dietary effects on the relative abundance of specific microbial taxa also differ by sex in humans and, to a lesser extent, in mice (172). Sex-specific differences in microbial composition and richness have also been reported in humans over the age of 60 and aged mice (163, 165, 166, 173).

## Functional Significance of Sex Differences in Immune Responses among Aged Individuals

### Vaccine Responses

In aged individuals, sex differences in antibody responses to vaccines are less consistent and depend on the vaccine antigen. The influence of sex and age has been most well studied for inactivated influenza virus vaccines as they are administered annually. For example, among individuals 65+ years of age, hemagglutinin inhibition antibody titers to both the standard and high dose seasonal trivalent inactivated influenza (TIV) vaccine are significantly higher in aged females when compared with males (174). Because influenza virus vaccines are available on an annual basis, a greater number of exposures (i.e., the behavioral act of seeking out vaccination) combined with the slower decline in immunity that occurs in aged females (see above) may contribute to sex differences in the antibody response to the TIV vaccine. By contrast, aged males have higher antibody responses to the tetanus diphtheria and pertussis (Td/Tdap) vaccines as well as the 7-valent and 23-valent pneumococcal vaccines (175–179). There is an insufficient number of studies from which to draw conclusions to understand why sex differences in vaccine-induced antibody responses are higher in aged females than males for a viral vaccine (i.e., the TIV vaccine), but lower in females than males for bacterial vaccines (i.e., the Td/Tdap and pneumococcal vaccines). If more vaccine studies were designed with *a priori* hypotheses about sex differences in vaccine-induced immunity, then we could begin to understand discrepancies in the findings following exposure to differential vaccine antigens.

Adverse reactions to vaccines, which are typically mild to moderate, can include both local (i.e., at the site of vaccination) and systemic reactions. Adverse reactions are reported by aged women more than their male counterparts in response to the seasonal and pandemic influenza vaccines (180–188), the pneumococcal vaccines (189, 190), the herpes zoster vaccine (191), or the tetanus and pertussis vaccines (192–194). While the types of adverse reactions experienced by aged males and females are typically similar, the proportion of females reporting redness, swelling, and injection site pain locally as well as headache, fever, chills, joint or muscle pain, headache, back and abdominal pain, or hypersensitivity reactions systemically is often greater than males. The prevailing hypothesis for differences in adverse reactions among aged males and females is that this reflects a gender-based reporting bias.

The efficacy of a vaccine is measured by the percent reduction in disease incidence in a vaccinated population (195). Sex-specific differences in vaccine efficacy are rarely considered, with most data coming from studies of influenza vaccines. Vaccine efficacy, which is defined by hospitalization and mortality rates post-vaccination, is lower in aged females than males, at least for the influenza vaccine (196–200). For other vaccines that are not administered annually, including the pneumococcal and herpes zoster vaccines, there are considerably less data. Overall, the efficacy both the herpes zoster and pneumococcal vaccines tends to be higher in aged females than their male counterparts (191, 201, 202).

### Autoimmunity

Most autoimmune patients are diagnosed between the ages of 20 and 60 years (203). For those whose autoimmune disease develops later, the disease tends to be milder and more easily controlled (203). Women are disproportionately affected by autoimmune disease, and this holds true for several autoimmune diseases with late-adult onset as well, including rheumatoid arthritis, polymyalgia rheumatica, and giant cell arteritis (Table 2). Regardless of the age of onset, the cellular and molecular basis of autoimmunity is complicated and distinct for each specific disease [reviewed in Ref. (204)]. Here, we focus on the impact of age and sex on autoimmune conditions with late onset.

**Table 2:** The female-to-male patient ratio for select mid-adult and late-adult onset autoimmune diseases.

Autoimmune disease	Autoimmune target	Mean age of onset (range) years	Female:male ratio	Reference
**Mid-adult onset**				
Multiple sclerosis	Myelin sheath	37 (25–45)	1.8:1	(205)
Myasthenia gravis	Neuromuscular junction	40	2.7:1	(205)
Systemic lupus erythematosus	Nuclear contents (systemic)	40 (30–50)	9:1	(205, 206)
Neuromyelitis optica	Optic nerve/spinal cord	32.6–45.7	2.4:1 ratio highest after age 65	(207, 208)
Graves’ disease	Thyroid	48	7.3:1	(205)
Systemic sclerosis	Connective tissue (systemic)	50 (35–65)	11.5:1	(209)

**Late-adult onset**				
Granulomatosis with polyangiitis (GPA) (formerly Wegener’s granulomatosis)	Cytoplasmic contents of neutrophils (systemic, vascular)	55 (40–70)	1:1 M > F after age 70	(205)
Rheumatoid arthritis	Joints	58 (42–74)	3:1	(210)
Polymyalgia rheumatica	Selected muscle groups	70–80	2.3:1	(211)
Giant cell arteritis	Vascular system	70–80	2.3:1	(211)

Although several theories have been proposed to explain sex differences in the cellular and molecular basis of aging [reviewed in Ref. (212)], perhaps most relevant to the sex-specific development of autoimmunity in the aged is that estrogen upregulates the activity of several antioxidant systems (213, 214). Dramatic loss of estrogen (such as during menopause) could be expected to result in increased cell death due to unchecked ROS-induced DNA damage. Indeed, fewer lymphocytes are detected in the blood of post-menopausal women compared with younger women (160, 215) and T cell apoptosis increases after natural or surgical menopause (216). This could especially explain increased female incidence of autoimmune diseases that may occur as a result of lymphopenia-induced homeostatic proliferation in the aged, although more studies are needed to test this hypothesis.

In mice, lymphopenia and the subsequent homeostatic proliferation of lymphocytes has been shown to contribute to the development of autoimmunity in many contexts [reviewed in Ref. (217)]. Certainly, there is an association between autoimmunity and lymphopenia in humans, but a strong case has not been made that lymphopenia is causative, or even occurs prior to, the onset of autoimmunity (218–224). However, evidence gathered by the laboratories of Goronzy et al. support a model whereby accelerated T cell loss in the aged, either due to telomerase deficiency, disruption to DNA repair responses, or menopause, may be sufficient to enable autoreactive T cells already present in the pool to respond to low-affinity self-antigens in rheumatoid arthritis patients [reviewed in Ref. (225)]. First, there is evidence of accelerated aging, or increased homeostatic proliferation in RA patients. The telomeres of naïve and memory T cells isolated from RA patients are shorter than age-matched controls (226) and T cell receptor diversity is reduced as well (227). Moreover, T cells from RA patients are more prone to apoptosis and are less capable of repairing dsDNA breaks (228). Finally, end-differentiated effector T cells that may be the consequence of homeostatic proliferation appear to be major participants in late onset autoimmune pathogenesis (229–232).

### Cancer

Sex and age influence cancer incidence and mortality, but the specific effects vary by cancer type. It is widely accepted that the probability of developing cancer increases with age (233). Although few studies have examined cancer incidence in those with very advanced age, it seems that cancer prevalence actually declines for those over the age of 85 (234, 235). There is some evidence to indicate that tumors may also be generally less aggressive in the extremely aged (236). Indeed, breast and prostate cancer patients over the age of 55 are more likely to develop tumors with characteristics associated with favorable treatment and/or survival outcomes (237, 238). However, it is not clear that tumors associated with other types of cancer, including bladder cancer, lung cancer, and acute myeloid leukemia, are indolent in older patients (239–242).

Overall, young men generally experience higher rates of cancer incidence and mortality than women (243–245). At advanced ages, men continue to experience higher incidences of most types of cancers, especially colorectal cancer, when compared with women (245, 246), but relative cancer mortality rates between older men and women differ by the particular cancer. Mortality differences between men and women diminish with age (especially after the age of 70) for colorectal cancer, stomach cancer, and leukemia (247). However, the male-to-female mortality ratio for brain cancer and myeloma decreases after middle age, but then increases again after the age of 70 (247).

The loss of sex hormones (especially due to menopause in women), age-associated immunosuppression, and chronic inflammation may contribute to sex- and age-specific patterns of cancer incidence and mortality. Indeed, the male preponderance of cancer incidence and mortality before menopause has been at least partially attributed to the protective effect of estrogen (248), presumably due to its ability to enhance immunosurveillance, as well as tissue-specific effects (249, 250). Purim et al. suggests that it takes 20–25 years for some cancers (such as colorectal) to develop and since changes in sex-specific incidence ratios for those cancers occur approximately 25 years after menopause, the loss of estrogens at approximately age 55 contributes to increased female cancer incidence after the age of 80 (246). On the other hand, age- and sex-related diminishment of the effectiveness of the immune system may not contribute a great deal to increased cancer incidence in the aged, since the types of cancers observed in the aged are not the same of those observed in immunocompromised patients. HIV-induced immunodeficiency is associated with lymphoma and Kaposi’s sarcoma, while most age-related malignancies in the aged are carcinomas (251). Finally, older persons with chronic inflammation may demonstrate increased risk of cancer, as it is clear that inflammation induced by viruses, bacteria, tobacco smoke, and obesity increases cancer risk (252–255). Overall, more studies are certainly warranted to better understand the factors that contribute to cancer incidence and mortality in older men and women.

#### Cancer Immunotherapy

Cancer immunotherapy trials typically involve younger patients with no co-morbidities, even though these characteristics are not representative of most cancer patients (256). This is particularly important because the effectiveness and dose of any particular immunotherapy is likely to be affected by age-associated changes in immunity and metabolism (256). In addition, few clinical trials are designed to compare the efficacy and safety of cancer immunotherapies between women and men of any age (257). The currently available data regarding the sex- and age-specific effectiveness of several immunotherapies are discussed below.

Checkpoint blockade therapies in young or middle-aged men and women appear to be beneficial, but the benefits may be stronger in men (258–261). Blockade of PD1/PDL1 with nivolumab was more effective in male melanoma and renal cell carcinoma patients than in female patients (258, 260). However, these studies were not designed to compare efficacy in male versus female patients, and the sample size for female patients was small. Preclinical studies of anti-PDL1 treatment revealed that melanoma tumor growth was more robustly reduced in female mice when compared with males (262). Estrogen upregulates PD-1 on T_regs_ and T_effs_. The authors speculated that anti-PDL1 treatment was more effective in females because of the greater contribution of PD-1 to T_reg_ suppression of antitumor responses in females (262). In addition, as mentioned above, the microbiome varies with age and sex and has recently been shown to significantly influence cancer immunotherapy success. Indeed, recent antibiotic use and the absence of specific microbial taxa correlates with reduced efficacy of PD1/PDL1 blockade and certain immune-reliant chemotherapies in both humans and mice [reviewed in Ref. (170, 263)]. Therefore, it is critical to more formally evaluate the effect of cancer immunotherapies in men and women and to assess the suitability of various cancer models for predicting the success of particular immunotherapies in the sexes.

As already mentioned, few clinical immunotherapy trials enroll patients of advanced age and studies that did include older patients reach different conclusions about the efficacy of checkpoint blockade in the aged. Meta-analyses of heterogeneous groups of cancer patients over the age of 65 or 70 treated with immune checkpoint inhibitors (biologicals targeting PD1, PDL1, or CTLA4) compared with similarly aged patients enrolled in the control arm of the studies revealed that checkpoint inhibitors reduced the risk of death by 34–37% in patients with advanced age (264, 265). Moreover, in at least one meta-analysis, the overall survival rate of patients over the age of 65 or 70 and younger patients treated with immune checkpoint inhibitors did not differ (264). However, other studies have reported significantly worse overall survival rates in patients over the age of 75 treated with checkpoint inhibitors (266). Finally, there is concern that treatment of older cancer patients with checkpoint inhibitors could actually enhance tumor growth, as occurred in one subset of cancer patients (267) or prompt immune-related adverse events, as occurs in mouse models (268).

## Conclusion

For most, the aging process is accompanied by alterations in the function of the immune system. Many experience chronic inflammation and a general impairment of immune cell function. The immune systems of young men and women are quite different, and it appears that aging affects the cellular composition and function of the immune system in sex-specific ways as well. This is likely because of pre-existing differences in immunity between men and women as well as differences in how menopause and andropause unfold. Age- and sex-specific changes to immunity may have consequences for late-adult onset autoimmunity and cancer, as well as for the efficacy of vaccinations and cancer immunotherapies. However, our understanding of the ways in which sex and age intersect with immune function and the consequences of this for autoimmunity, cancer, and therapeutic interventions is severely limited by the lack of inclusion of these variables in clinical and preclinical studies. Therefore, preclinical and clinical studies related to vaccination, autoimmunity, and cancer therapies must be powered to detect sex effects, in accordance with the sex and gender equity in research (SAGER) guidelines (269). Age, sex hormone concentrations, hormone replacement therapies, and health status must be considered as well, given the known impact of these variables on immune-related conditions common in the aged (Table 3).

**Table 3:** Variables to consider when designing clinical studies related to immunity in the aged.

	Clinical study considerations
Age	Clearly defined age categories
	Young: 20 to ≤45 years
	Old: >45 to ≤85 years
	Very old/elderly: >85 years

Health status	Frailty: three of the five following characteristics: weight loss, weakened handgrip, exhaustion, reduced gait speed, and reduced activity
	Concentrations of serum inflammatory proteins: IL-6, TNF-α, IL-1β, and C-reactive protein

Sex hormone status	Time of menopause
	Serum concentrations of sex hormones
	Hormone replacement therapy

## Author Contributions

MB conceived of the idea for this review. MB and SK outlined the content. MB, TP, AF, and SK researched and wrote sections. All authors edited and reviewed the final draft.

## Conflict of Interest Statement

The authors declare that the research was conducted in the absence of any commercial or financial relationships that could be construed as a potential conflict of interest.
